# Perturbations in cardiac metabolism in a human model of acute myocardial ischaemia

**DOI:** 10.1007/s11306-021-01827-x

**Published:** 2021-08-23

**Authors:** Sanoj Chacko, Mamas A. Mamas, Magdi El-Omar, David Simon, Sohaib Haseeb, Farzin Fath-ordoubadi, Bernard Clarke, Ludwig Neyses, Warwick B. Dunn

**Affiliations:** 1 Division of Cardiology, Queen’s University, Kingston, ON Canada; 2 Institute of Cardiovascular Sciences, Manchester Academic Health Sciences Centre, University of Manchester, Manchester, UK; 3 Keele Cardiovascular Research Group, Keele University, Stoke-on-Trent, UK; 4 Manchester Heart Centre, Manchester Royal Infirmary, Central Manchester University Hospitals NHS Trust, Manchester, UK; 5 Department of Chemistry, Queen’s University, Kingston, ON Canada; 6 School of Chemistry and Manchester Centre for Integrative Systems Biology, Manchester Institute of Biotechnology, The University of Manchester, Manchester, UK; 7 University of Luxembourg, 4365 Esch-sur-Alzette, Luxembourg; 8 School of Biosciences and Institute of Metabolism and Systems Research, University of Birmingham, Birmingham, UK; 9 Kingston Health Sciences Centre, Queen’s University, 76 Stuart St, Kingston, ON Canada

**Keywords:** Acute myocardial ischemia, Coronary sinus serum, Metabolomics, Metabolism, PCI

## Abstract

**Introduction:**

Acute myocardial ischaemia and the transition from reversible to irreversible myocardial injury are associated with abnormal metabolic patterns. Advances in metabolomics have extended our capabilities to define these metabolic perturbations on a metabolome-wide scale.

**Objectives:**

This study was designed to identify cardiac metabolic changes in serum during the first 5 min following early myocardial ischaemia in humans, applying an untargeted metabolomics approach.

**Methods:**

Peripheral venous samples were collected from 46 patients in a discovery study (DS) and a validation study (VS) (25 for DS, 21 for VS). Coronary sinus venous samples were collected from 7 patients (4 for DS, 3 for VS). Acute myocardial ischaemia was induced by transient coronary occlusion during percutaneous coronary intervention (PCI). Plasma samples were collected at baseline (prior to PCI) and at 1 and 5 min post-coronary occlusion. Samples were analyzed by Ultra Performance Liquid Chromatography-Mass Spectrometry in an untargeted metabolomics approach.

**Results:**

The study observed changes in the circulating levels of metabolites at 1 and 5 min following transient coronary ischaemia. Both DS and VS identified 54 and 55 metabolites as significant (P < 0.05) when compared to baseline levels, respectively. Fatty acid beta-oxidation and anaerobic respiration, lysoglycerophospholipids, arachidonic acid, docosahexaenoic acid, tryptophan metabolism and sphingosine-1-phosphate were identified as mechanistically important.

**Conclusion:**

Using an untargeted metabolomics approach, the study identified important cardiac metabolic changes in peripheral and coronary sinus plasma, in a human model of controlled acute myocardial ischaemia. Distinct classes of metabolites were shown to be involved in the rapid cardiac response to ischemia and provide insights into diagnostic and interventional targets.

**Supplementary Information:**

The online version contains supplementary material available at 10.1007/s11306-021-01827-x.

## Introduction

Cardiovascular disease (CVD) is the leading cause of death in men and women, accounting for approximately 30% of deaths worldwide (Lopez et al., 2006). The Global Burden of Disease study in 2013 estimated that 17.3 million deaths worldwide were related to CVD (GBD 2013 Mortality & Causes of Death Collaborators, 2015). Acute coronary syndrome (ACS) represents a spectrum of events ranging from unstable angina (UA) and non-ST-elevation myocardial infarction (NSTEMI) to ST-elevation myocardial infarction (STEMI), depending on whether there is sustained occlusion of the coronary artery by thrombus (STEMI), or whether this is transient (NSTEMI). NSTEMI is considered to have occurred if biomarkers of myocardial injury such as cardiac troponin (cTn) or the MB fraction of creatine kinase (CKMB) have been released (Mythili & Malathi, 2015). The diagnosis of an ACS has traditionally included a combination of ischaemic symptoms, electrocardiogram (ECG) changes, and elevations in serum biomarkers (Adams et al., 1993; Thygesen et al., 2018). However, the symptoms can be atypical or absent in many patients. ECG changes that aid in the early diagnosis may be non-specific or even absent in around 40% of the patients (Rouan et al., 1989). As a result, early diagnosis of ACS can pose a challenge and relies largely upon serum biomarkers, preferably cTn, due to its myocardial tissue specificity and their dynamic pattern (Eggers et al., 2004). Detection of a rise and /or fall of cTn value with at least one value above the 99^th^ percentile upper reference limit, when measured at baseline and 3–6 h after clinical presentation, is considered an essential criterion for the diagnosis of ACS (Thygesen et al., 2012). Myocardial ischaemia and its consequent metabolic alteration is a crucial stage in the continuum of ACS which can result in myocyte injury, dysfunction, and potential fatal dysrhythmia (Morrow et al., 2001; Opie, 1993). Identifying these rapid changes in the metabolic pattern may reveal important pathophysiologic mechanisms and potential targets for intervention to provide a favorable equilibrium.

Metabolic phenotyping, otherwise known as metabolomics, is a rapidly growing field in systems biology that provides the ability to monitor rapid and dynamic metabolic changes in human biofluids and tissues (Dunn et al., 2011a, 2011b; Holmes et al., 2008). Plasma, a frequently examined pool of metabolites, has been the main source for metabolic profiling (Fan et al., 2016). The application of metabolomics to different models of myocardial injury is an emerging field. Metabolomic analysis of serum has been used to predict exercise-inducible ischemia (Barba et al., 2008; Sabatine et al., 2005). The utility of metabolic profiling has also been demonstrated under controlled conditions of myocardial ischemia (Lin et al., 2009). Recently, the predictive value of metabolomics in identifying patients at risk of in-stent restenosis (ISR) following PCI was demonstrated. Metabolites belonging to phospholipid and sphingolipid metabolism classified patients with ISR and control subjects with a sensitivity and specificity of 91% and 90%, respectively (Cui et al., 2017). NMR-based metabolomics have also been used to identify patients at risk of adverse outcomes and death within two years following ACS (Vignoli et al., 2019). Much progress has been made in the use of metabolomics to identify biomarkers which may aid in the early detection and prognosis of ACS (Liu et al., 2020; Pouralijan Amiri et al., 2019; Yao et al., 2017). The metabolic profiling of peripheral blood samples from 27 patients with UA and 15 healthy subjects identified hexadecanoic acid, octadecanoic acid, 1-deoxyglucose and D-ribofuranose, with 80% precision prediction for patients with unstable angina (UA) (Shi et al., 2011). Similarly, another study of 45 patients with UA and 43 asymptomatic patients with atherosclerosis as controls identified phospholipids, including phytosphingosines, phosphatidylcholines and phosphatidylglycerols to be significantly up-regulated in patients with UA when compared to controls (Sun et al., 2013). In a more recent multicenter prospective study, metabolomic profiling was conducted to identify the metabolic signatures associated with future risk of recurrence of angina following PCI. The study identified eicosanoids, sphingolipids and acylcarnitines as prognostic metabolites in predicting patients at risk of recurrence of angina (Cui et al., 2021).

The aim of this study was to evaluate myocardial tissue-specific metabolic changes in peripheral and coronary sinus serum in the first 5 min following acute myocardial ischaemia in humans.

## Materials and methods

### Ethical approval and consent procedures

This study complies with the Declaration of Helsinki and was approved by the Manchester Research Ethics Committee (Ref: 04/Q1406/58) and Central Manchester and Manchester Children’s University Hospitals NHS Trust Research and Development (PIN:10562). Eligible patients were contacted and provided with patient study information sheet. Patients were given sufficient time (minimum 24 h), prior to the clinic appointment. The study was discussed during the clinic visit when the patients have opportunity to clarify any concerns and accept or decline to participate in the study. A written consent was obtained for all those who expressed their wish to participate in the study.

### Study population and surgical procedure

This study was based in a tertiary care centre at The Manchester Heart Centre and Central Manchester University Hospitals National Health Service (NHS) Foundation Trust. The study population included a carefully selected cohort of stable elective patients with single-vessel coronary artery disease. Patients with coronary artery bypass surgery, recent acute coronary syndrome (less than six weeks from the procedure), triple vessel coronary artery disease, left ventricular systolic dysfunction, structural heart disease, anaemia, renal impairment, chronic inflammatory conditions, and malignancy were excluded.

Blood samples were obtained from 46 patients undergoing elective percutaneous coronary intervention (PCI) to native coronary arteries over a period of 8 months. Peripheral venous samples were collected through a venous sheath inserted into the femoral vein. Coronary sinus sampling was performed in 7 patients following cannulation of the coronary sinus using a 6 Fr Amplatz Left-1 catheter (AL-1) during the procedures. All the PCI procedures were performed via the femoral artery. Most of the participants agreed for the peripheral femoral venous sample collection rather than coronary sinus sampling due to the less-invasive nature of the procedure.

Control samples of venous blood were obtained either from the coronary sinus or via the sheath in the femoral vein once the guide catheter and guide wire were in position before initiation of the PCI procedure (defined as baseline). Myocardial ischaemia was generated during the initial balloon inflation to pre-dilate the target lesion during PCI. Balloon inflations of between 30 s and 1 min were performed until ECG changes, ST segment depression or ST segment elevation and patient symptoms, such as chest tightness or pain consistent with myocardial ischaemia were noted. Individual operators decided upon the type and size of the angioplasty balloon and inflation pressures that were used to pre-dilate the target lesions. Serial venous samples were collected from either the coronary sinus or femoral vein at 1 min (defined as TP1) and 5 min (defined as TP5). PCI was then completed as per routine.

Patient demographics and procedural data were collected at the time of intervention. Samples were collected into EDTA Vacutainer tubes (Becton Dickinson, Franklin Lakes, New Jersey) and transported on ice for immediate centrifugation at 1500×*g* for 15 min at 4 °C. Plasma was then removed and stored in aliquots at − 80 °C. Two studies were undertaken, defined as the Discovery Study (DS) and the Validation Study (VS). The process for assignment to the DS or VS groups was based on the time of patient recruitment with the first set of patients recruited being assigned to the DS group (patients 1–25) and the remaining set of patients recruited being assigned to the VS group (patients 26–46). Peripheral and coronary sinus blood was collected from 21 and 4 subjects, respectively, in the DS and from 18 and 3 subjects in the VS, respectively. Peripheral blood data for one subject in the DS group was identified as an outlier (the number of metabolite features detected was less than 35% that of the mean of metabolite features detected across all samples) and was removed from the dataset following data acquisition.

### Definitions

The epicardial coronary blood vessels consists of left main coronary artery branching into left anterior descending (LAD) and circumflex (LCx) coronary arteries and the right coronary artery (RCA). A coronary atherosclerotic lesion of 70% or more is considered significant requiring coronary revascularization. The American College of Cardiology/American Heart Association (ACC/AHA) task force have classified the coronary atherosclerotic lesions as A, B and C. for predicting success and complications during coronary interventions (Ryan et al., 1988). The lesion was defined as type A if it was discrete, non-angulated, or non-calcified lesion with no major branch involvement. Type B lesions were tubular, eccentric, moderately angulated, calcified, with bifurcation involvement or ostial location. Type C lesions were diffuse, extremely tortuous, calcified, and total occlusion for more than 3 months. Based on the classification, there is an association of slightly decreasing procedural success and increasing procedural risk with lesions staging from A to C.

### Metabolic phenotyping

All reagents used were of HPLC grade purity (Sigma-Aldrich CHROMASOLV, Dorset, United Kingdom). Plasma samples were extracted applying liquid–liquid extraction as follows: 200 μL aliquots of plasma were mixed with 600 μL of methanol using a vortex mixer (15 s) followed by centrifugation (13,000×*g*, 15 min). Supernatants were transferred to Eppendorf tubes and lyophilized (HETO VR MAXI vacuum centrifuge attached to a HETO CT/DW 60E cooling trap; Jouan, Gydevang, Denmark). A single pooled quality control (QC) sample (intra-study QC sample) was prepared by pooling 200 μL aliquots of plasma from each sample followed by vortex mixing for 60 s. Twenty-two (DS) and nineteen (VS) 200 μL aliquots of the intra-study QC sample were transferred to separate Eppendorf tubes for extraction as described above for the plasma samples.

All samples were analyzed separately in positive and negative ion modes using Ultra Performance Liquid Chromatography-Mass Spectrometry (UPLC-MS) (Waters Acquity UPLC system coupled to a Thermo LTQ-Orbitrap XL) applying a previously described method (Dunn et al., 2011a, 2011b). Raw data was processed applying the open-source software XCMS as previously described (Dunn et al., 2011a, 2011b) to construct a data matrix for positive ion mode data and a data matrix for negative ion mode data. Signal correction was performed to minimize run-order associated drift in response by applying quality control-based robust LOESS (locally estimated scatterplot smoothing) signal correction (QC-RLSC) (Dunn et al., 2011a, b). Quality assessment and quality-based filtering of the data were performed applying the data acquired for QC samples only. The relative standard deviation (RSD) for QC samples only (from QC sample injection nine onwards) and the percentage of QC samples where a response was reported were calculated for each metabolite feature. Data for all biological samples and QC samples for all metabolites with a relative standard deviation for response > 20% in the studied QC samples and after QC-RLSC were removed from the dataset prior to statistical analysis. Data for all biological samples and QC samples for all metabolites which were detected in < 60% of the studied QC samples were removed from the dataset prior to statistical analysis. The Wilcoxon signed-rank test was applied to peripheral blood sample data only to identify statistically significant metabolites when comparing baseline to TP1 samples and baseline to TP5 samples. Metabolites were annotated applying the software PUTMEDID_LCMS (Brown et al., 2011) to level 2 of the Metabolomics Standards Initiative reporting guidelines for chemical analysis (Sumner et al., 2007). Further work to identify several metabolites based on MS/MS spectra was also performed with matching to an internal retention time and MS/MS library developed by Dunn et al. (2011a, 2011b) or to mzCloud (https://www.mzcloud.org/). Metabolites were grouped into ‘metabolite classes’ based on chemical structure similarity (e.g. fatty acids) or metabolic pathway similarity (e.g. TCA cycle). Box and whisker plots were constructed with MetaboAnalyst using normalized data (Chong et al., 2019). Pathway enrichment analysis was performed using MetaboAnalyst (pathway analysis, compound name list inputted, visualization method = scatter plot, enrichment method = hypergeometric, topology analysis = relative betweenness centrality, pathway library = homo sapiens (KEGG)).

Experimental workflow was identical for samples taken from the DS and VS. The metabolomics data acquisition for the DS and VS was performed in October 2010 and August 2011, respectively.

## Results

### Population demographics

The study population consisted of a cohort of 46 patients; 25 patients in the DS and 21 patients in the VS. The patient characteristics, lesion data, and procedural data are shown in Table 1. In both groups, the majority of participants were male and Caucasian. There was a large difference in the vessel diameter (range 2.50–2.99 mm) between the DS and VS. Apart from this, there were no significant differences in the target vessels or lesion types between the studies.

**Table 1:** Demographics and clinical profile of the study population

Baseline variables	DS (n = 25)	VS (n = 21)	p value
Age (years)	63.9 ± 9.0	63.0 ± 9.0	
Male	84%	85.7%	
Caucasians	88%	90.4%	
Risk factors			0.674
Hypertension	12/25 (48%)	11/21 (52%)	
Diabetes	15/25 (60%)	7/21 (33.3%)	
Hyperlipidemia	22/25 (88%)	19/21 (90.4%)	
Smoking	10/25 (40%)	8/21 (38%)	
BMI (kg/m^2^)	27.84 ± 5.35	26.47 ± 1.96	
Drugs			0.992
Antiplatelets	25/25 (100%)	21/21 (100%)	
Beta-blockers	20/25 (80%)	16/21 (76.2%)	
ACEi	17/25 (68%)	14/21 (66.6%)	
Statins	25/25 (100%)	21/21 (100%)	
Nitrates	6/25 (24%)	4/21 (19%)	
Calcium channel blockers	6/25 (24%)	7/21 (33.3%)	
Lesion characteristics			0.777
LAD	16/25 (64%)	12/21 (57.1%)	
RCA	6/25 (24%)	7/21 (33.3%)	
Cx	3/25 (12%)	2/21 (9.5%)	
Vessel diameter (mm)			**0.017**
2.50–2.99	11/25 (44%)	1/21 (4.8%)	
3.00–3.49	7/25 (28%)	8/21 (38%)	
3.50–3.99	6/25 (24%)	8/21 (38%)	
4.50–5.00	1/25 (4%)	4/21 (19%)	
Lesion length (mm)			0.993
10–14	4/25 (16%)	4/21 (19%)	
15–19	5/25 (20%)	5/21 (23.8%)	
20–24	8/25 (32%)	6/21 (28.6%)	
25–30	5/25 (20%)	4/21 (19%)	
> 30	3/25 (12%)	2/21 (9.5%)	
Lesion type			0.657
A	3/25 (12%)	1/21 (4.8%)	
B	10/25 (40%)	10/21 (47.6%)	
C	12/25 (48%)	10/21 (47.6%)	
% Stenosis			0.713
50–74	3/25 (12%)	2/21 (9.5%)	
75–94	15/25 (60%)	15/21 (71.4%)	
> 95	7/25 (28%)	4/21 (19%)	
Procedural data			
Mean Ischemic period	31.1 ± 1.87	31.6 ± 3.65	
Median ST elevation	0.5 mm	0.5 mm	
Median ST depression	0.5 mm	0.5 mm	
Mean troponin	0.04 ng/ml	0.03 ng/ml	
Mean CK	102.3 U/L	97.3 U/L	

*BMI* body mass index, *ACEi* angiotensin-converting enzyme inhibitor, *Cx* circumflex, *CK* creatine kinase, *DS* discovery study, *LAD* left anterior descending, *RCA* right coronary artery, *VS* validation study

Bold defines p < 0.05

Pre-dilatation of the target lesions was performed with angioplasty balloons inflated between 14 to 22 atmospheres (mean 15 atmospheres) for a period of between 30 to 60 s (mean 31.1 s). During balloon inflation, ischemic ECG changes associated with chest discomfort were noted in 26/46 patients (56.5%), with ST-segment elevation in 19/46 patients (41.3%), and ST-segment depression in 7/46 patients (15.2%). In the remaining 20/46 patients (43.4%), there were no ECG changes observed, although 11/20 patients (55.0%) reported transient chest discomfort during this period. A total of 9 (out of 46) patients did not have ECG changes or symptoms during the procedure in whom balloon inflation was performed up to the maximum period of 60 s to create transient ischaemia. The PCI procedure was then completed in all cases, and no major adverse events and complications were documented up to 30 days’ post-procedure.

### Metabolomic phenotyping

The metabolic composition of peripheral plasma samples collected at baseline, TP1, and TP5 was obtained using UPLC-MS. There were 2692 and 1012 metabolite features (*m/z*-RT pairs) detected in positive and negative ion modes, respectively, after quality assessment and data filtering in the DS. There were 3163 and 919 metabolite features detected in positive and negative ion modes, respectively, after the same process in the VS. There were 432 and 480 unique metabolites annotated in negative ion mode in the DS and VS, respectively, with 179 unique metabolites identified in both the DS and VS studies. There were 1093 and 1143 unique metabolites annotated in positive ion mode in the DS and VS, respectively, with 384 unique metabolites identified in both the DS and VS studies. Following statistical analysis, 108 and 203 unique metabolites were identified as differing (p < 0.05) in the DS and VS, respectively, when comparing baseline and TP1 samples; of these, 54 unique metabolites were identified as differing (p < 0.05) in TP1 (Supplementary Table S1). Similarly, 117 and 214 unique metabolites were identified as differing (p < 0.05) in the DS and VS, respectively, when comparing baseline and TP5 samples; of these, 55 metabolites were identified as statistically significant in both studies (Supplementary Table S2). There were 30 and 77 unique metabolites identified as differing (p < 0.05) in the DS and VS, respectively, when comparing TP1and TP5 samples; of these, nine metabolites were identified as statistically significant in both studies (Supplementary Table S3).

**Fig. 1:**
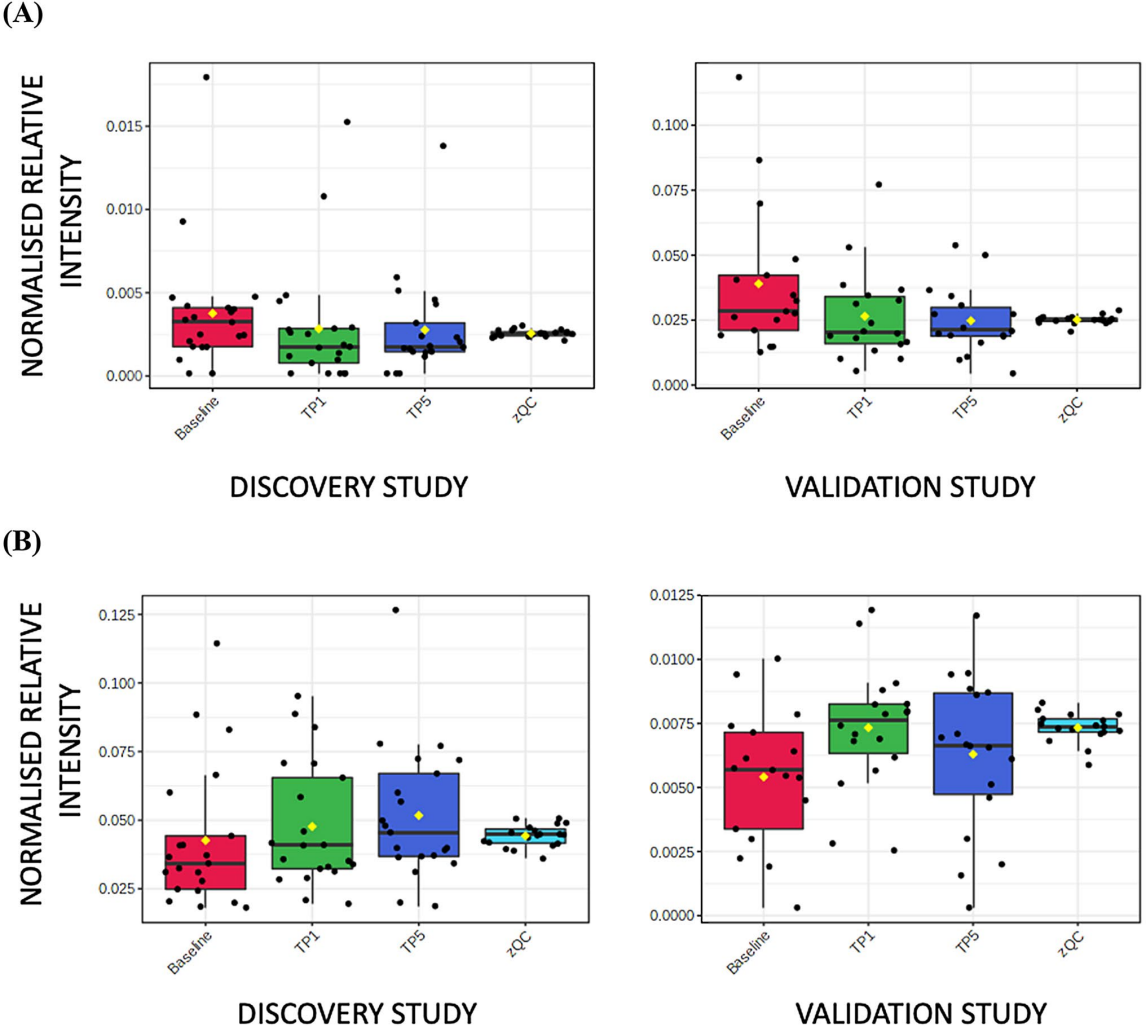
Box and whisker plot describing normalized relative intensity values in the concentration of octanoyl carnitine (**A**) and hexadecanoic acid (**B**) at three timepoints (0 = baseline, 1 = TP1 and 5 = TP5) for peripheral samples. QC sample data are included (QC). Data for the discovery (left) and validation (right) studies are shown (p < 0.05)

**Fig. 2:**
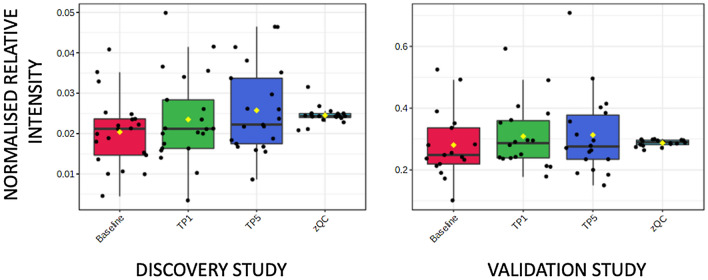
Box and whisker plot describing normalized relative intensity values in the concentration of LysoPC(20:4) at three timepoints (0 = baseline, 1 = TP1 and 5 = TP5) for peripheral samples. QC sample data are included (QC). Data for the discovery (left) and validation (right) studies are shown (p < 0.05)

**Fig. 3:**
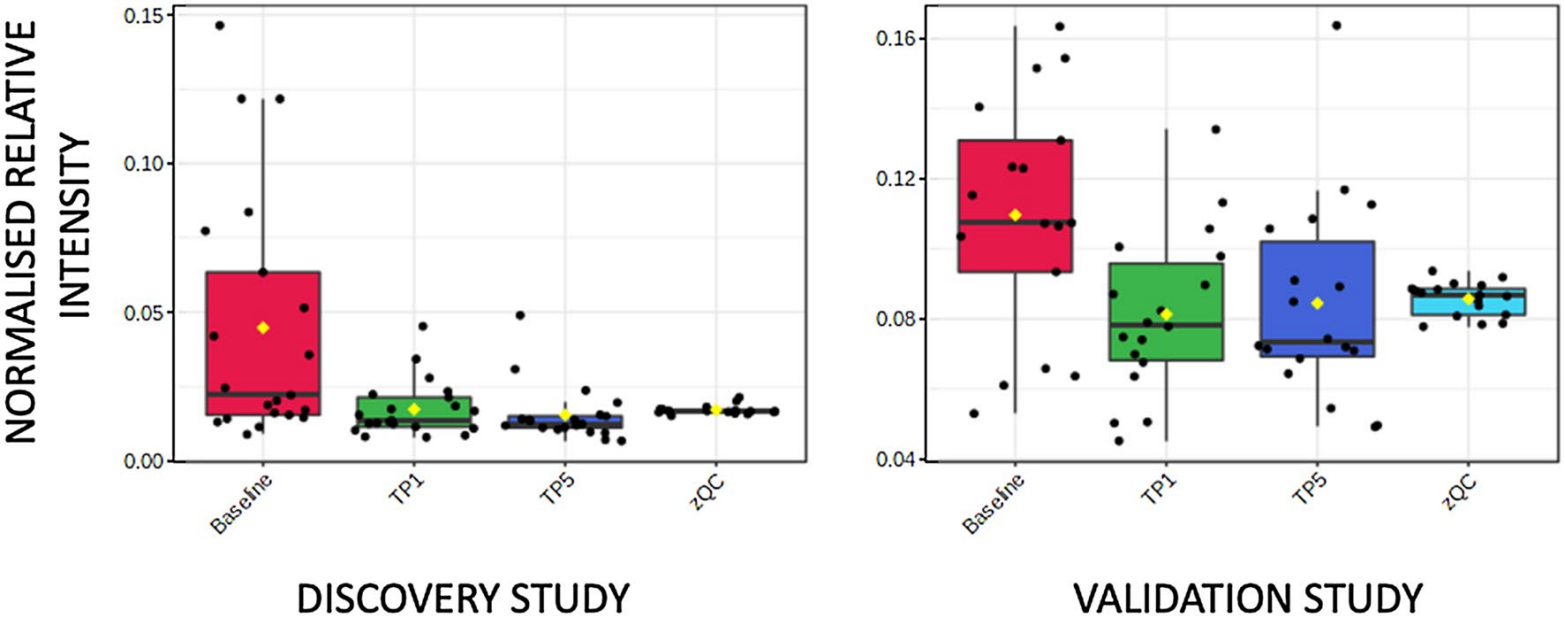
Box and whisker plot describing normalized relative intensity values in the concentration of tryptophan at three timepoints (0 = baseline, 1 = TP1 and 5 = TP5) for peripheral samples. QC sample data are included (QC). Data for the discovery (left) and validation (right) studies are shown

**Fig. 4:**
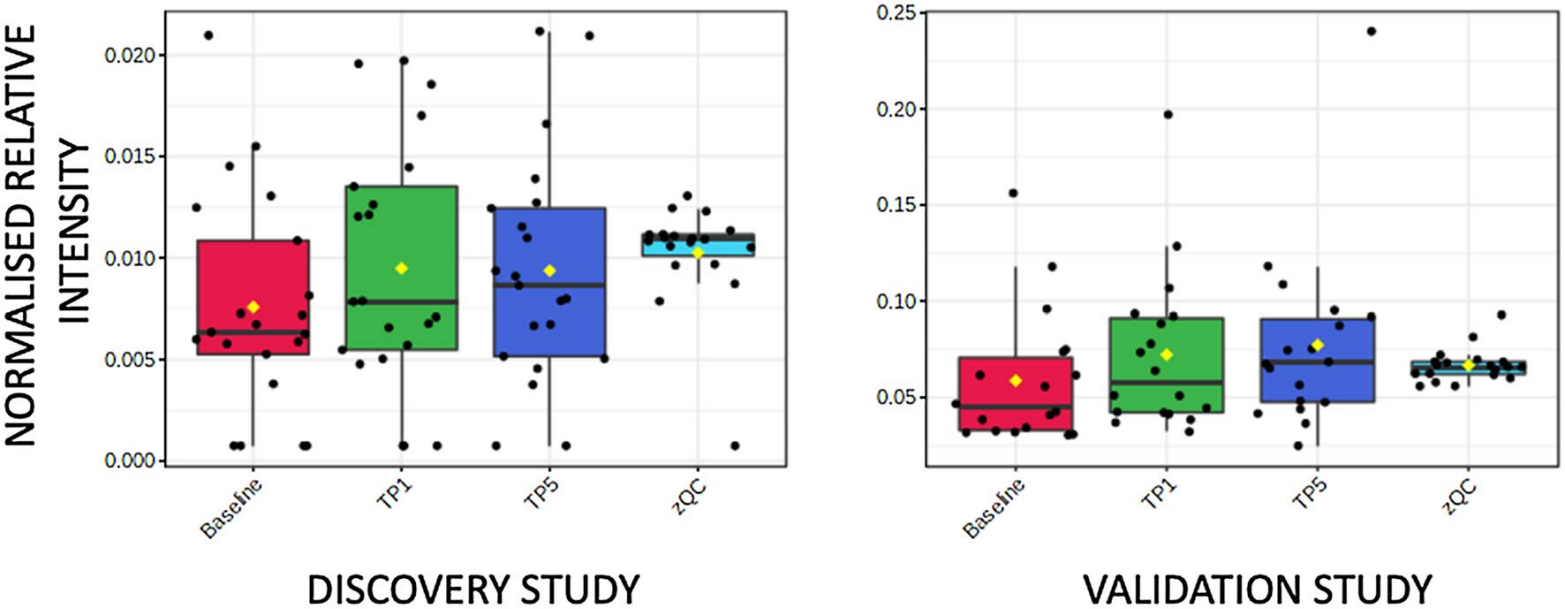
Box and whisker plot describing normalized relative intensity values in the concentration of arachidonic acid at three timepoints (0 = baseline, 1 = TP1 and 5 = TP5) for peripheral samples. QC sample data are included (QC). Data for the discovery (left) and validation (right) studies are shown (p < 0.05)

Supplementary Tables S1 and S2 highlight classes of metabolites where more than one metabolite showed a change in both DS and VS. Two acyl carnitines (octanoyl carnitine and decanoyl carnitine) showed higher concentrations in baseline samples compared to both TP1 and TP5 samples. Eleven and twelve fatty acids showed either higher or lower concentrations in baseline samples compared to both TP1 and TP5 samples in the DS and VS, respectively. Boxplots for two examples are shown in Fig. 1A (octanoyl carnitine) and Fig. 1B (hexadecanoic acid). C6–C14 fatty acids showed a higher concentration at baseline when compared to TP1 and TP5, and C16–C22 fatty acids showed a higher concentration at TP1 and TP5 when compared to baseline. In addition, eight and twelve lysoglycerophospholipids showed higher concentrations in TP1 and TP5 samples when compared to baseline samples in the DS and VS, respectively (Fig. 2). Two diacylglycerides showed a higher concentration at baseline samples when compared to TP1 and TP5 samples. Other changes were observed in aromatic metabolites, including tryptophan, with six and seven metabolites showing a change in the DS and VS, respectively (Fig. 3). These metabolites showed both increases and decreases in their concentration when comparing baseline to TP1 and TP5 samples. Six short-chain fatty acids also showed a statistically significant difference when comparing baseline to TP1 and TP5 samples in both studies with increases and decreases in their concentration when comparing baseline to TP1 and TP5 samples. Arachidonic acid showed a significant upregulation at TP1 and TP5 in both sample types (peripheral and coronary sinus) after transient coronary ischemia (Fig. 4).

Robust changes were observed in these and other metabolite classes in both the DS and VS. Acyl carnitines (3 and 3 metabolites), aromatic metabolites (15 and 20 metabolites), bile acids (6 and 7 metabolites), carbohydrates (7 and 12 metabolites), diacylglycerides (7 and 6 metabolites), fatty acids (38 and 31 metabolites), lysoglycerophospholipids (25 and 20 metabolites), purine and pyrimidine metabolites (7 and 5 metabolites), short-chain organic acids (4 and 5 metabolites), sterols and steroids (12 and 11 metabolites), vitamin D metabolites (6 and 8 metabolites), TCA metabolites (2 and 1 metabolites) and branched-chain amino acid metabolism (2 and 1 metabolite) all showed differences in one or both of the DS and VS, respectively. Pathway enrichment analysis demonstrated that tryptophan metabolism was enriched in two timepoint comparisons, baseline vs. TP1 and baseline versus TP5.

The mechanistic relevance of metabolites observed as differing from baseline in both studies in relation to prior diagnosis of diabetes and hypertension, BMI, and ST elevation during the procedure was investigated. LysoPC (20:4) was higher across all time points in non-diabetic compared to diabetic patients (p < 0.05 for TP1). A metabolite annotated as 6-(alpha-D-glucosaminyl)-1D-myo-inositol or lactosamine was present at a lower concentration in subjects with BMI < 26 compared to subjects with BMI > 26 (p < 0.05 for TP5).

## Discussion

This study has used a metabolomics approach to quantify the changes in cardiac metabolite release in response to early myocardial ischaemia in humans. We analyzed blood samples from the peripheral and coronary sinus plasma in the first 5-min period after PCI and demonstrated that distinct classes of metabolites were involved in the rapid cardiac response to ischaemia. Several previous studies have demonstrated the application of metabolomics to delineate the patterns of cardiac metabolism in different models of myocardial injury (Barba et al., 2008; Lewis et al., 2008; Lin et al., 2009; Sabatine et al., 2005; Teul et al., 2011; Turer et al., 2009). However, their greatest limitations are, (i) the small number of targeted metabolites studied, and (ii) that most of the studies involved myocardial infarction with irreversible myocyte injury as opposed to early myocardial ischaemia, as investigated in the study reported here. Our study is interesting for several reasons. Firstly, our unbiased approach identified that the significant metabolic perturbations predominantly involved lipid metabolism during early cardiac ischaemia. Secondly, only minor fractions of the metabolome were altered during early cardiac ischaemia suggesting highly specific or highly regulated changes. Thirdly, metabolites which have previously been shown to be detrimental to cardiac function such as arachidonic acid, as well as metabolites which are known to have a cardioprotective effect such as docosahexaenoic acid, were identified as metabolically important following cardiac ischaemia, along with other metabolites previously not associated with acute ischaemia.

The balance between carbohydrate and fatty acid use as substrates in energy production is well reported in association with cardiac ischaemia (Li et al., 2012; Oliver, 2015; Pascual & Coleman, 2016; Stanley et al., 2005). Ischaemia reduces aerobic oxidation of fatty acids and increases anaerobic respiration of glucose for ATP synthesis. In our study, two acyl carnitines showed a decrease in concentration following the ischemic event in both the DS and VS with eleven and twelve fatty acids demonstrating either an increase or decrease in concentration after the ischaemic event. It is notable that the same changes were observed in coronary sinus samples and in peripheral blood samples. This implies that the metabolic changes are myocardial specific. C6-C14 fatty acids showed a decrease in concentration following an ischaemic event whereas C16-C22 fatty acids showed an increase in concentration. There were 7 and 6 diacylglycerides along with 38 and 31 fatty acids that showed differential concentrations in one of the two studies. These data show that there is an increase in the use or a decrease in the synthesis of short and medium chain fatty acids, and a decrease in the use or an increase in the synthesis of long chain and very long chain fatty acids for beta-oxidation. Although changes in carbohydrates have been previously reported, the analytical method applied is not appropriate for a detailed investigation of glucose metabolism, though some changes in carbohydrate metabolites were observed.

In our study, eight and twelve lysoglycerophospholipids were observed to increase in concentration following the ischaemic event in both studies with a further 25 and 20 lysoglycerophospholipids showing a statistically significant change in either the discovery or validation study, respectively. An example is shown in Fig. 2 and the same changes were observed in coronary sinus samples and in peripheral blood samples implying that the metabolic changes are myocardial specific. Plasma phospholipase A2 cleaves a single fatty acid from glycerophospholipids and has been shown to increase in the ischaemic myocardium (Leong et al., 1992). Lysoglycerophosphocholines (LysoPC) have been reported to increase in a model of myocardium starvation and ischemia and are markers for incident coronary heart disease (Ganna et al., 2014). The modulation of arachidonic acid release has been shown to be regulated by LysoPC levels in rat heart myoblastic H9c2 cells with an enhanced release of LysoPCs associated with palmitoyl (C16:0) or stearoyl (C18:0) groups (Golfman et al., 1999). The accumulation of LysoPCs has also been linked to dysrhythmia following ischaemia (Sobel et al., 1978). Arachidonic acid has been shown to increase following an ischaemic event (van der Vusse et al., 1982), and in our study population, we found that there was significant up-regulation at TP1 and TP5 in both sample types (peripheral and coronary sinus) after transient coronary ischaemia. Arachidonic acid is known to have a detrimental effect on cardiac function including through potassium channel activation (Kim & Clapham, 1989), leukocyte maintenance (Mullane et al., 1987), and reactive oxygen species production (Cocco et al., 1999). A number of studies have found a positive correlation between the amount of arachidonic acid and the risk of coronary heart disease; for example, Kark et al. (2003) examined an Israeli population consuming a diet high in linoleic acid (LA), a precursor of arachidonic acid, and reported a relation between risk of ACS and adipose tissue arachidonic acid (though not LA) (Kark et al., 2003).

In our study, tryptophan metabolism showed a decrease in concentration following an ischaemic event, with six and seven associated aromatic metabolites showing a statistically significant difference in both the DS and VS, respectively. Fifteen and 20 aromatic metabolites showed a difference in only the DS or VS, respectively. This included tryptophan, N-formylkynurenine and kynurenine which all demonstrated a decrease in relative concentration at TP1 and TP5 compared to baseline for the VS study only. Histidine-Tryptophan-Ketoglutarate (HTK) solution is applied before heart transplantation for heart preservation, and higher concentrations of tryptophan have shown beneficial effects on outcome (Careaga et al., 2001; Lee et al., 2010; Saitoh et al., 2000).

Importantly, most metabolites shown to differ in their concentrations when comparing before and after an acute ischaemic event were not known to be associated with BMI, diabetes, hypertension or ST elevation during the procedure. This implies that the metabolites discussed above are dynamic responders to cardiac ischaemia independent of associated risk factors of CVD. Of the limited number of statistically significant changes, an unidentified metabolite showed changes related to BMI and ST elevation during PCI. Further studies may be warranted to further investigate the mechanistic basis of this observation.

## Conclusion

This discovery-based mass spectrometry study has defined myocardial tissue-specific metabolic changes related to acute myocardial ischaemia in the 5-min period after PCI, therefore representing rapid tissue-specific metabolic changes to the ischemic event. This study has investigated multiple different areas of metabolism simultaneously and has identified diverse yet specific areas of metabolism requiring further investigation.

### Limitations

There are a number of limitations to the study. It is possible that some of the changes in metabolites that we report to have occurred during transient coronary occlusion may relate to vascular trauma rather than ischemia itself. This is unlikely as low pressure balloon inflations were employed, which are not associated with significant vascular trauma (personal intra-vascular imaging observation from MAM). Nevertheless, we cannot entirely rule out this possibility.

## Supplementary Information

Below is the link to the electronic supplementary material.

Supplementary file1 (DOCX 54 kb)
