# Supplementary material for: Perturbations in cardiac metabolism in a human model of acute myocardial ischaemia
Source: Metabolomics. 2021 Aug 23;17(9):76. doi: 10.1007/s11306-021-01827-x (PMC8382649; doi:10.1007/s11306-021-01827-x)
Supplement: Supplementary file 1 — Supplementary file1 (DOCX 54 kb) [file 11306_2021_1827_MOESM1_ESM.docx]

**Supplementary Material**

**Supplementary Table S1:** Metabolites defined as statistically significant (p<0.05) when comparing baseline and TP1 samples in both the discovery and validation studies. FC is fold change, calculated applying the median relative concentrations at baseline/TP1.

**Supplementary Table S2:**  Metabolites defined as statistically significant (p<0.05) when comparing baseline and TP5 samples in both the discovery and validation studies. FC is fold change, calculated applying the median relative concentrations at baseline/TP5.

**Supplementary Table S3:**  Metabolites defined as statistically significant (p<0.05) when comparing TP1 and TP5 samples in both the discovery and validation studies. FC is fold change, calculated applying the median relative concentrations at TP1/TP5.

**Supplementary Table S1**

|  |  | **Discovery study** | | **Validation study** | |
| --- | --- | --- | --- | --- | --- |
| **Metabolite** | **Metabolite Class** | **p.value** | **FC** | **p.value** | **FC** |
| Octanoylcarnitine | Acyl carnitine | 0.00628 | 1.34 | 0.00005 | 1.41 |
| Decanoylcarnitine | Acyl carnitine | 0.01269 | 1.23 | 0.00038 | 1.43 |
| Tryptophan | Aromatic metabolites | 0.00039 | 2.68 | 0.00209 | 1.32 |
| 2',6'-Dihydroxy-4'-methoxyacetophenone;"3-(2,3-Dihydroxyphenyl)propanoate";3-(3-hydroxyphenyl)-3-hydroxypropanoic acid;3-(4-Hydroxyphenyl)lactate;3-Methoxy-4-hydroxyphenylglycolaldehyde;Dihydrocaffeic acid;Homovanillate;Isohomovanillic acid;;Phenylacetic acid | Aromatic metabolites | 0.00048 | 1.93 | 0.00158 | 1.51 |
| Formyl-N-acetyl-5-methoxykynurenamine;Phenylacetylglutamine;N-Acetylserotonin;N-methyltryptophan | Aromatic metabolites | 0.00059 | 1.12 | 0.01823 | 1.40 |
| 2,5,6-Trihydroxy-5,6-dihydroquinoline;3-succinoylpyridine;adrenochrome;Hippurate;N-Acetylanthranilate | Aromatic metabolites | 0.01947 | 0.78 | 0.00665 | 0.75 |
| Hydroxybenzoate;Salicylate | Aromatic metabolites | 0.02664 | 0.96 | 0.00019 | 1.20 |
| 2-Carboxy-2,3-dihydro-5,6-dihydroxyindole;Hydroxyhippuric acid;Carboxyphenylglycine;Dopaquinone;Salicyluric acid | Aromatic metabolites | 0.03623 | 1.16 | 0.00281 | 1.15 |
| Galactose 6-sulfate;Glucose 6-sulfate | Carbohydrate | 0.00026 | 1.17 | 0.00042 | 1.41 |
| 1,5-Anhydro-D-glucitol;Deoxy-galactose;Deoxy-glucose;Rhamnose;Fucose;Fuculose;Rhamnulose | Carbohydrate | 0.03277 | 1.06 | 0.02081 | 1.32 |
| Phenylgalactoside | Carbohydrate | 0.03999 | 1.13 | 0.03849 | 0.89 |
| DG(38:6);DG(36:3) | Diacylglycerol | 0.02157 | 1.24 | 0.03954 | 1.30 |
| DG(32:2) | Diacylglycerol | 0.03277 | 1.03 | 0.00896 | 1.11 |
| Hydroxy-tetradecenoic acid;Oxo-tetradecanoic acid | Fatty acid | 0.00102 | 1.26 | 0.00769 | 1.15 |
| Heptenoic acid | Fatty acid | 0.00428 | 2.59 | 0.03479 | 1.14 |
| Methyl-octadecanoic acid;Nonadecanoic acid | Fatty acid | 0.00730 | 1.22 | 0.01743 | 0.34 |
| Hydroxy-oxo-hexadecanoic acid;Dihydroxycyclopentaneundecanoic acid;pentadecenoic acid | Fatty acid | 0.00944 | 0.71 | 0.01823 | 0.73 |
| Docosahexaenoic acid | Fatty acid | 0.01069 | 0.78 | 0.00336 | 0.68 |
| Eicosadienoic acid;Icosatrienoic acid | Fatty acid | 0.01362 | 0.78 | 0.02685 | 0.65 |
| Hexadecanoate | Fatty acid | 0.02148 | 0.85 | 0.00790 | 0.74 |
| Icosatetraenoic acid;Eicosatetraenoic acid | Fatty acid | 0.02664 | 0.76 | 0.01593 | 0.81 |
| Oxo-hexacosenoic acid | Fatty acid | 0.02664 | 1.26 | 0.00107 | 0.81 |
| Heptacosanoic acid | Fatty acid | 0.04405 | 1.06 | 0.01203 | 1.08 |
| Octadecadienoic acid | Fatty acid | 0.04599 | 0.86 | 0.01500 | 1.19 |
| LysoPC(22:6) | Lysoglycerophospholipid | 0.00072 | 0.81 | 0.00011 | 0.71 |
| LysoPC(20:2) | Lysoglycerophospholipid | 0.00233 | 0.75 | 0.01387 | 0.77 |
| LysoPC(22:5) | Lysoglycerophospholipid | 0.00365 | 0.72 | 0.00002 | 0.71 |
| LysoPC(18:2) | Lysoglycerophospholipid | 0.00486 | 0.88 | 0.00084 | 0.90 |
| LysoPC(18:1) | Lysoglycerophospholipid | 0.01208 | 0.89 | 0.03423 | 0.92 |
| LysoPC(20:4) | Lysoglycerophospholipid | 0.01531 | 0.84 | 0.00084 | 0.89 |
| LysoPC(20:5) | Lysoglycerophospholipid | 0.01947 | 0.64 | 0.00067 | 0.76 |
| LysoPC(16:0) | Lysoglycerophospholipid | 0.02395 | 0.84 | 0.02016 | 1.07 |
| MG(20:0) | Monoacylglyceride | 0.03999 | 1.15 | 0.00258 | 1.27 |
| 2-Acetamido-2-deoxy-6-O-a-L-fucopyranosyl-D-glucose;3-O-fucopyranosyl-2-acetamido-2-deoxyglucopyranose;N-Acetyl-6-O-L-fucosyl-D-glucosamine | Oligosaccharide | 0.02633 | 0.86 | 0.00066 | 0.77 |
| Aminoadenosine;Oxo-Adenosine;Dehydroadenosine;S-aminomethyldihydrolipoamide | Other class | 0.01531 | 1.31 | 0.00042 | 1.87 |
| 1,3-Dimethyl-6,8-isoquinolinediol | Other class | 0.01578 | 1.24 | 0.00790 | 1.24 |
| Prolylhydroxyproline | Peptide | 0.00628 | 2.21 | 0.01743 | 1.87 |
| 10-Deoxymethynolide | Polyketide | 0.00286 | 1.57 | 0.00209 | 1.27 |
| Hypoxanthine | Purine metabolite | 0.03506 | 1.80 | 0.00557 | 1.95 |
| 5-Amino-6-(5'-phosphoribosylamino)uracil | Riboflavin metabolism | 0.02664 | 1.09 | 0.01593 | 1.53 |
| 2-Methyl-3-ketovaleric acid;Oxohexanoic acid | Short chain organic acids | 0.00021 | 1.29 | 0.00008 | 1.20 |
| Ethylhydracrylic acid;Hydroxy-methylbutyric acid;Hydroxyvaleric acid | Short chain organic acids | 0.00039 | 1.56 | 0.01203 | 1.15 |
| Hydroxybutanoic acid | Short chain organic acids | 0.00048 | 0.82 | 0.00665 | 0.50 |
| Oxohexanoic acid;Methyl-oxopentanoate | Short chain organic acids | 0.01578 | 1.22 | 0.02016 | 1.36 |
| Butenoate;Isocrotonic acid | Short chain organic acids | 0.02157 | 0.78 | 0.00258 | 0.75 |
| Lactate | Short chain organic acids | 0.02958 | 1.06 | 0.03479 | 1.14 |
| Sphingosine 1-phosphate | Sphingolipids | 0.04844 | 1.15 | 0.01099 | 1.31 |
| 11beta,21-Dihydroxy-3,20-oxo-5beta-pregnan-18-al;16alpha-Hydroxycorticosterone;"17alpha,21-Dihydroxy-5beta-pregnane-3,11,20-trione";18-Hydroxycorticosterone;"1alpha,17alpha,21-trihydroxy-20-oxo-22,23,24,25,26,27-hexanorvitamin D3;"4,5alpha-Dihydrocortisone";Cortisol | Sterol and steroid metabolism | 0.01923 | 1.12 | 0.00896 | 1.38 |
| (20S)-1alpha,20,25-trihydroxy-24a-homovitamin D3;"(20S)-1alpha,25-dihydroxy-20-methoxyvitamin D3;"(23R)-1alpha,23,25-trihydroxy-23-methylvitamin D3;"(24R)-1alpha,24-dihydroxy-26,27-dimethyl-22-oxavitamin D3;"(24S)-1alpha,24-dihydroxy-26,27-dimethyl-22-oxavitamin D3;13'-carboxy-gamma-tocopherol;"1alpha,25-dihydroxy-11alpha-(hydroxymethyl)vitamin D3;"1alpha,25-dihydroxy-11alpha-methoxyvitamin D3;"1alpha,25-dihydroxy-11beta-methoxyvitamin D3;"1alpha,25-dihydroxy-24a,24b-dihomo-22-oxa-20-epivitamin D3;"1alpha,25-dihydroxy-24a,24b-dihomo-22-oxavitamin D3;"1alpha,25-dihydroxy-24a,24b-dihomo-23-oxa-20-epivitamin D3;"1alpha,25-dihydroxy-24a,24b-dihomo-23-oxavitamin D3;"1alpha,25-dihydroxy-26,27-dimethyl-20,21-didehydro-23-oxavitamin D3;"1alpha,25-dihydroxy-26,27-dimethyl-22-oxavitamin D3;"1alpha-hydroxy-18-(4-hydroxy-4-methylpentyloxy)-23,24,25,26,27-pentanorvitamin D3;(22S)-22-hydroxyvitamin D3;(24R)-24-hydroxyvitamin D3;(24S)-24-hydroxyvitamin D3;(5E)-1alpha-hydroxy-3-epivitamin D3;(5E)-1alpha-hydroxyvitamin D3;(5E)-1beta-hydroxy-3-epivitamin D3 ;(5E)-1beta-hydroxyvitamin D3 | Vitamin D metabolism | 0.00944 | 1.16 | 0.00934 | 1.12 |
| (22R)-1alpha,22,25-trihydroxy-26,27-dimethyl-23,24-tetradehydro-24a-homo-20-epivitamin D3;"(22R)-1alpha,25-dihydroxy-22-methoxy-26,27-dimethyl-23,23,24,24-tetradehydrovitamin D3;"(22S)-1alpha,22,25-trihydroxy-26,27-dimethyl-23,23,24,24-tetradehydro-24a-homovitamin D3;"(22S)-1alpha,22,25-trihydroxy-26,27-dimethyl-23,24-tetradehydro-24a-homo-20-epivitamin D3;"(22S)-1alpha,25-dihydroxy-22-methoxy-26,27-dimethyl-23,24-tetradehydro-20-epivitamin D3;"1alpha-hydroxy-18-(4-hydroxy-4-ethyl-2-hexynyloxy)-23,24,25,26,27-pentanorvitamin D3 | Vitamin D metabolism | 0.01947 | 0.72 | 0.01743 | 1.09 |
| 24-Hydroxygeminivitamin D3;1alpha,25-dihydroxy-2beta-(5-hydroxypentoxy)vitamin D3 | Vitamin D metabolism | 0.02664 | 1.08 | 0.00557 | 1.17 |
| (20S)-1alpha,25-dihydroxy-20-methoxy-26,27-dimethylvitamin D3;"(22R)-1alpha,22,25-trihydroxy-26,27-dimethyl-24a-homo-20-epivitamin D3;"(22S)-1alpha,22,25-trihydroxy-26,27-dimethyl-24a-homovitamin D3;"1alpha,25-dihydroxy-26,27-dimethyl-24a,24b-dihomo-22-oxa-20-epivitamin D3;"1alpha,25-Dihydroxy-2alpha-(3-hydroxypropyl)vitamin D3";"1alpha,25-dihydroxy-2beta-(3-hydroxypropyl)vitamin D3;"1alpha-hydroxy-18-(4-hydroxy-4-ethylhexyloxy)-23,24,25,26,27-pentanorvitamin D3;1alpha-hydroxy-2beta-(3-hydroxypropoxy)vitamin D3;"26,27-diethyl-1alpha,25-dihydroxy-22-oxavitamin D3;"26,27-diethyl-1alpha,25-dihydroxy-23-oxavitamin D3 ;(6R)-6,19-ethano-25-hydroxy-6,19-dihydrovitamin D3;"(6S)-6,19-ethano-25-hydroxy-6,19-dihydrovitamin D3;"1alpha-hydroxy-26,27-dimethylvitamin D3;1-Hydroxyvitamin D5;"25-hydroxy-26,27-dimethylvitamin D3;"25-Hydroxy-6,19-dihydro-6,19-ethanovitamin D3 | Vitamin D metabolism | 0.03999 | 1.08 | 0.00934 | 1.26 |

**Supplementary Table S2**

|  |  | **Discovery study** | | **Validation study** | |
| --- | --- | --- | --- | --- | --- |
| **Metabolite** | **Metabolite Class** | **p.value** | **FC** | **p.value** | **FC** |
| Octanoylcarnitine | Acyl carnitine | 0.00628 | 1.27 | 0.00029 | 1.51 |
| Decanoylcarnitine | Acyl carnitine | 0.00902 | 1.28 | 0.00021 | 1.53 |
| Tryptophan | Aromatic metabolites | 0.00013 | 1.60 | 0.00011 | 2.59 |
| 2',6'-Dihydroxy-4'-methoxyacetophenone;"3-(2,3-Dihydroxyphenyl)propanoate";3-(3-hydroxyphenyl)-3-hydroxypropanoic acid;3-(4-Hydroxyphenyl)lactate;3-Methoxy-4-hydroxyphenylglycolaldehyde;Dihydrocaffeic acid;Homovanillate;Isohomovanillic acid;;Phenylacetic acid | Aromatic metabolites | 0.00121 | 2.12 | 0.00033 | 1.57 |
| 2,5,6-Trihydroxy-5,6-dihydroquinoline;3-succinoylpyridine;adrenochrome;Hippurate;N-Acetylanthranilate | Aromatic metabolites | 0.00488 | 0.76 | 0.00790 | 0.75 |
| 2-Amino-5-phosphopentanoic acid;2-Methylquinoline-3,4-diol;3-Hydroxy-2-methyl-1H-quinolin-4-one;3-Indoleglycolaldehyde;"3-Methyl-quinolin-2,8-diol";5-Hydroxyindoleacetaldehyde;Indole-3-acetate;Indoleacetic acid | Aromatic metabolites | 0.00710 | 1.18 | 0.00557 | 1.29 |
| Methylquinoline-3,4-diol;3-Hydroxy-2-methyl-1H-quinolin-4-one;3-Indoleglycolaldehyde;"3-Methyl-quinolin-2,8-diol";5-Hydroxyindoleacetaldehyde;Gentianine;Indole-3-acetate;Indoleacetic acid;Naphthoquinone | Aromatic metabolites | 0.00801 | 1.15 | 0.00934 | 1.30 |
| 1,3-Dimethyl-6,8-isoquinolinediol | Aromatic metabolites | 0.01578 | 1.17 | 0.00258 | 1.37 |
| Mandelate;2-(Hydroxymethyl)benzoic acid;2-Hydroxyphenylacetate;"3,4-Dihydroxyphenylacetaldehyde";3-Hydroxyphenylacetate;3-Methoxytropolone;3-Methylsalicylate;4-Hydroxy-3-methoxy-benzaldehyde;4-Hydroxy-3-methylbenzoic acid;4-Hydroxymethylsalicylaldehyde;4-Hydroxyphenacyl alcohol;4-Hydroxyphenyl acetate;4-Hydroxyphenylacetate;4-Methoxybenzoate;4-Methylsalicylate;6-Methylsalicylate;isovanillin;Phenoxyacetate | Aromatic metabolites | 0.02148 | 2.72 | 0.00336 | 11.28 |
| 2-Carboxy-2,3-dihydro-5,6-dihydroxyindole;Hydroxyhippuric acid;Carboxyphenylglycine;Dopaquinone;Salicyluric acid | Aromatic metabolites | 0.02385 | 1.20 | 0.00233 | 1.13 |
| Galactose 6-sulfate;Glucose 6-sulfate | Carbohydrate | 0.00730 | 1.16 | 0.00002 | 1.50 |
| DG(38:6);DG(36:3) | Diacylglyceride | 0.00186 | 1.39 | 0.00066 | 1.37 |
| DG(38:5);DG(36:2) | Diacylglyceride | 0.04208 | 1.19 | 0.00029 | 1.28 |
| Hydroxydecanedioic acid | Fatty acid | 0.00013 | 2.91 | 0.03954 | 1.44 |
| hydroxy-oxo-hexadecanoic acid;Dihydroxycyclopentaneundecanoic acid;pentadecenoic acid | Fatty acid | 0.00026 | 0.62 | 0.00002 | 0.61 |
| Docosahexaenoic acid | Fatty acid | 0.00071 | 0.72 | 0.02322 | 0.83 |
| Icosatetraenoic acid;Eicosatetraenoic acid | Fatty acid | 0.00121 | 0.74 | 0.03423 | 0.76 |
| Hydroxyhexanoic acid | Fatty acid | 0.00639 | 1.28 | 0.02685 | 1.09 |
| Hydroxystearate | Fatty acid | 0.00944 | 0.69 | 0.02367 | 0.71 |
| Hexadecanoate | Fatty acid | 0.01069 | 0.81 | 0.04828 | 0.74 |
| Eicosadienoic acid;Icosatrienoic acid | Fatty acid | 0.01208 | 0.73 | 0.03037 | 0.64 |
| hydroxy-hexadecanoic acid | Fatty acid | 0.01718 | 0.79 | 0.03423 | 0.89 |
| hydroxy-tetradecenoic acid;Oxo-tetradecanoic acid | Fatty acid | 0.03192 | 1.48 | 0.01041 | 1.18 |
| Dodecanedioic acid;hendecenoic acid;undecenoic acid | Fatty acid | 0.03623 | 0.85 | 0.04317 | 0.81 |
| Eicosapentaenoic acid;Icosapentaenoic acid | Fatty acid | 0.04844 | 0.73 | 0.02081 | 0.62 |
| LysoPC(20:2) | Lysoglycerophospholipid | 0.00000 | 0.73 | 0.00896 | 0.77 |
| LysoPC(20:4) | Lysoglycerophospholipid | 0.00004 | 0.78 | 0.00665 | 0.87 |
| LysoPC(18:2) | Lysoglycerophospholipid | 0.00013 | 0.84 | 0.00233 | 0.91 |
| LysoPC(22:5) | Lysoglycerophospholipid | 0.00026 | 0.77 | 0.00029 | 0.78 |
| LysoPC(22:6) | Lysoglycerophospholipid | 0.00048 | 0.76 | 0.00008 | 0.75 |
| LysoPC(18:1) | Lysoglycerophospholipid | 0.00059 | 0.85 | 0.00233 | 0.86 |
| LysoPC(16:0) | Lysoglycerophospholipid | 0.00121 | 0.77 | 0.00557 | 1.11 |
| LysoPC(20:5) | Lysoglycerophospholipid | 0.00143 | 0.77 | 0.00042 | 0.76 |
| LysoPC(O-16:0/0:0) | Lysoglycerophospholipid | 0.00639 | 0.88 | 0.03479 | 1.14 |
| LysoPC(17:1) | Lysoglycerophospholipid | 0.00831 | 0.78 | 0.04317 | 0.92 |
| LysoPC(16:1) | Lysoglycerophospholipid | 0.01208 | 0.86 | 0.00042 | 0.88 |
| LysoPC(dm16:0);LysoPC(O-16:1/0:0) | Lysoglycerophospholipid | 0.01718 | 0.90 | 0.01286 | 1.14 |
| 1-O-alpha-D-glucopyranosyl-(2-hexadecanoyloxy)-eicosan-1-ol | Other class | 0.04208 | 1.63 | 0.01743 | 1.47 |
| 16-Hydroxyestrone;estradiol-2,3-quinone;estrone-2,3-semiquinone | Other class | 0.04208 | 1.25 | 0.01743 | 1.52 |
| beta-Alanine;Alanine;Sarcosine;Methylglyoxal | Other class | 0.04599 | 1.11 | 0.00464 | 1.16 |
| Prolylhydroxyproline | Peptide | 0.00375 | 1.74 | 0.00790 | 2.07 |
| gamma-glutamyl-L-isoleucine;gamma-glutamyl-L-leucine | Peptide | 0.02958 | 1.39 | 0.01823 | 1.34 |
| 10-Deoxymethynolide | Polyketide | 0.00801 | 1.52 | 0.01286 | 1.24 |
| Hypoxanthine | Purine | 0.00100 | 1.82 | 0.00385 | 2.17 |
| Dimethylguanosine | Purine | 0.00554 | 1.31 | 0.02322 | 1.15 |
| Dimethylxanthine;Theobromine;Theophylline | Purine | 0.01755 | 1.09 | 0.02667 | 1.20 |
| Hydroxybutanoic acid | Short chain organic acid | 0.00001 | 0.76 | 0.00658 | 0.81 |
| Ethylhydracrylic acid;Hydroxy-methylbutyric acid;Hydroxyvaleric acid | Short chain organic acid | 0.00005 | 1.65 | 0.01387 | 1.19 |
| 2-Methyl-3-ketovaleric acid;Oxohexanoic acid;Methyl-oxopentanoate | Short chain organic acid | 0.00186 | 1.34 | 0.00001 | 1.23 |
| dimethyl-butenoic acid;methyl-pentenoic acid;hexenoic acid | Short chain organic acid | 0.00248 | 0.63 | 0.03052 | 0.79 |
| Butenoate;Isocrotonic acid | Short chain organic acid | 0.01135 | 0.77 | 0.00168 | 0.71 |
| Oxohexanoic acid;Methyl-oxopentanoate | Short chain organic acid | 0.01417 | 1.21 | 0.01500 | 1.34 |
| 4-Sulfobenzaldehyde | Sulphur metabolism | 0.00944 | 0.82 | 0.00067 | 0.40 |
| (22R)-1alpha,22,25-trihydroxy-26,27-dimethyl-23,24-tetradehydro-24a-homo-20-epivitamin D3;"(22R)-1alpha,25-dihydroxy-22-methoxy-26,27-dimethyl-23,23,24,24-tetradehydrovitamin D3;"(22S)-1alpha,22,25-trihydroxy-26,27-dimethyl-23,23,24,24-tetradehydro-24a-homovitamin D3;"(22S)-1alpha,22,25-trihydroxy-26,27-dimethyl-23,24-tetradehydro-24a-homo-20-epivitamin D3;"(22S)-1alpha,25-dihydroxy-22-methoxy-26,27-dimethyl-23,24-tetradehydro-20-epivitamin D3;"1alpha-hydroxy-18-(4-hydroxy-4-ethyl-2-hexynyloxy)-23,24,25,26,27-pentanorvitamin D3 | Vitamin D metabolism | 0.00375 | 0.62 | 0.04477 | 1.06 |
|  |  |  |  |  |  |

**Supplementary Table S3**

|  |  | **Discovery study** | | **Validation study** | |
| --- | --- | --- | --- | --- | --- |
| **Metabolite** | **Metabolite Class** | **p.value** | **FC** | **p.value** | **FC** |
| 1alpha-hydroxy-26,27-dinorvitamin D3 25-carboxylic acid | Vitamin D metabolism | 0.0192 | 1.3 | 0.0067 | 1.23 |
| 24-Nor-5beta-chol-22-ene-3alpha,7alpha,12alpha-triol | Bile acid metabolism | 0.0459 | 1.26 | 0.0001 | 1.18 |
| DG(36:4);DG(34:1) | Diacylglyceride | 0.0459 | 1.11 | 0.0267 | 1.14 |
| Heneicosanedioic acid;MG(18:1) | Other class | 0.0121 | 1.14 | 0.0267 | 1.28 |
| LysoPC(18:1) | Lysoglycerophospholipid | 0.0083 | 0.95 | 0.009 | 0.92 |
| LysoPC(18:2) | Lysoglycerophospholipid | 0.0172 | 0.95 | 0.015 | 1.07 |
| methyl-hexadecanedioic acid;Heptadecanedioic acid | Fatty acid | 0.0484 | 0.89 | 0.00007 | 1.2 |
| MG(14:0) | Monoacylglyceride | 0.0025 | 1.34 | 0.00001 | 1.22 |
| MG(16:0) | Monoacylglyceride | 0.001 | 2.01 | 0.0046 | 1.14 |
